# Exploring the Relationship Between the Contents of a Goal-Setting Diary and Goal Attainment: A Foundational Study for Healthy Individuals

**DOI:** 10.7759/cureus.82543

**Published:** 2025-04-18

**Authors:** Katsuma Ikeuchi, Hisanori Fukunaga, Ryutaro Aoki, Masayuki Takagi, Yusuke Imamoto

**Affiliations:** 1 Department of Occupational Therapy, Faculty of Health and Welfare, Prefectural University of Hiroshima, Mihara, JPN; 2 Department of Occupational Therapy, Yamato University Hakuho Junior College Division, Kitakatsuragi, JPN; 3 Department of Occupational Therapy, Shimane Rehabilitation College, Nita, JPN

**Keywords:** developing tool, foundational study, goal-setting, multiple regression analysis, occupational therapy, self-monitoring

## Abstract

Introduction: The goal-setting diary is designed to enhance goal-related performance through self-monitoring. It is essential to ensure that the contents of the diary are related to the degree of goal attainment and simplify its format prior to clinical application. This foundational study aimed to investigate the relationship between the contents of a goal-setting diary and goal attainment.

Methods: The participants were 252 healthy occupational therapy students (OTS) aged 18 years or older from three training institutions (a university, a junior college, and a vocational school). Students with diseases that are being treated were excluded. Multiple regression analysis (stepwise method) was performed, with goal attainment as the dependent variable. Quantitative data from the goal-setting diary, including the number of strategies, importance, satisfaction, confidence to continue, difficulty, persistence, self-efficacy, and effort, along with the participant’s basic attributes, were used as explanatory variables.

Results: A total of 167 healthy participants completed a goal-setting diary for seven days (response rate: 66.3%). Multiple regression analysis revealed effort (B = 0.58, p < 0.001, 95% confidence interval, 0.46-0.71) and satisfaction (B = 0.05, p < 0.001, 95% confidence interval, 0.03-0.06) as significant explanatory variables for goal attainment (adjusted R^2^ = 0.57).

Conclusions: Our results emphasize the importance of daily monitoring of effort and satisfaction levels in relation to goal attainment. Furthermore, the goal-setting diary can be simplified because it has many sections. As the participants in this study were healthy OTS, future studies are needed to apply the revised goal-setting diary, which is composed of effort and satisfaction, to clients in clinical settings and examine its effectiveness.

## Introduction

Goal-setting is a formal process in which a rehabilitation professional or team, in collaboration with the patient and/or their family, negotiates goals [1]. Although current person-centered goal-setting interventions encourage active engagement, only about half of these interventions use self-evaluation or assess progress toward goal achievement [2]. Self-management programs, such as self-monitoring, goal-setting, and problem-solving, are beneficial as they contribute to improved quality of life and self-efficacy [3]. Therefore, integrating a self-monitoring strategy into rehabilitation programs is important.

The goal-setting theory proposed by Locke and Latham is one of the most commonly used theories. The core statement of this theory is that setting specific, challenging goals (“goal core” indicating the nature of goals) and committing to these goals positively impact individual performance levels [4]. Therefore, the implications of this theory are considered relevant to goal-setting in the context of occupations, activities, and participation [5].

“Moderators” and “mechanisms” play key roles in the relationship between the “goal core” and the performance to attain the goals [4]. “Moderators” indicate the conditions for maximizing performance, including goal importance, confidence to continue activities related to goals, and task difficulty. “Mechanisms” refer to the effects of setting specific goals, including the efforts made to attain goals, strategies, persistence, and self-setting (whether the goals were set by oneself or others). Furthermore, the satisfaction resulting from performance and goal achievement reinforces the moderators [4]. Specifically, goals are more likely to be attained when they are perceived as important and the tasks are difficult, as these factors encourage greater effort toward goal achievement. Consequently, enhanced satisfaction further improves performance.

Although the goal-setting theory has been developed in the field of organizational psychology [4], it has also been applied to the field of rehabilitation in recent years. We conducted a literature review to explore factors affecting individual performance in rehabilitation practice based on the goal-setting theory [5]. We then designed a goal-setting diary that allows people to self-monitor and engage in high-quality activities related to their goals.

Current research based on the goal-setting diary is limited to a case report [6], which showed that a survivor of cancer who set the goal of “finding leisure activities” was able to identify and enjoy meaningful leisure activities. Given the theoretical framework [4,5] and this case report [6], it can be inferred that using the goal-setting diary to monitor daily activities supports goal attainment. This suggests the potential for future applications in rehabilitation practice. However, it is essential to validate the goal-setting diary itself, ensuring that its content influences goal achievement, and simplify the diary, which currently includes many sections, for practical use. Hence, this study uses multiple regression analysis to explore the relationship between the contents of the goal-setting diary and the degree of goal attainment.

## Materials and methods

Participants and sample sizes

The participants in this study were 252 healthy occupational therapy students (OTS) aged 18 years or older from three training institutions (a university, a junior college, and a vocational school). Students with diseases that are being treated were excluded. At the time of data collection, 118 OTS (46.8%) were enrolled in the university, 74 (29.4%) in the junior college, and 60 (23.8%) in the vocational school. These training institutions did not offer courses on the goal-setting theory.

The sample size required to calculate the multiple correlation coefficient using multiple regression analysis is ≥50 + 8 × m, where m is the number of explanatory variables [7]. This study included 10 explanatory variables (detailed below); hence, we needed to obtain data from 130 OTS. In a previous survey for OTS and physical therapy students (n = 102), the response rate was 75% [8]. However, the response rate for this study was expected to be approximately 60% because the goal-setting diary needed to be completed within a certain period. Therefore, data were collected from three training institutions.

Data collection

Goal-Setting Diary

**Figure 1:**
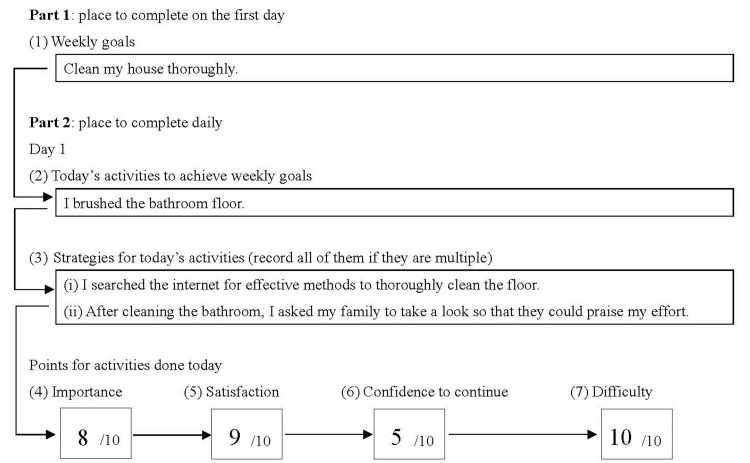
A sample of the sections included in the goal-setting diary Although Part 2 encompasses seven days, only the first day is shown in this figure. The participants recorded in boxed sections (1)-(7). When writing the scores in (4)-(7), they referred to the Likert scale

**Figure 2:**
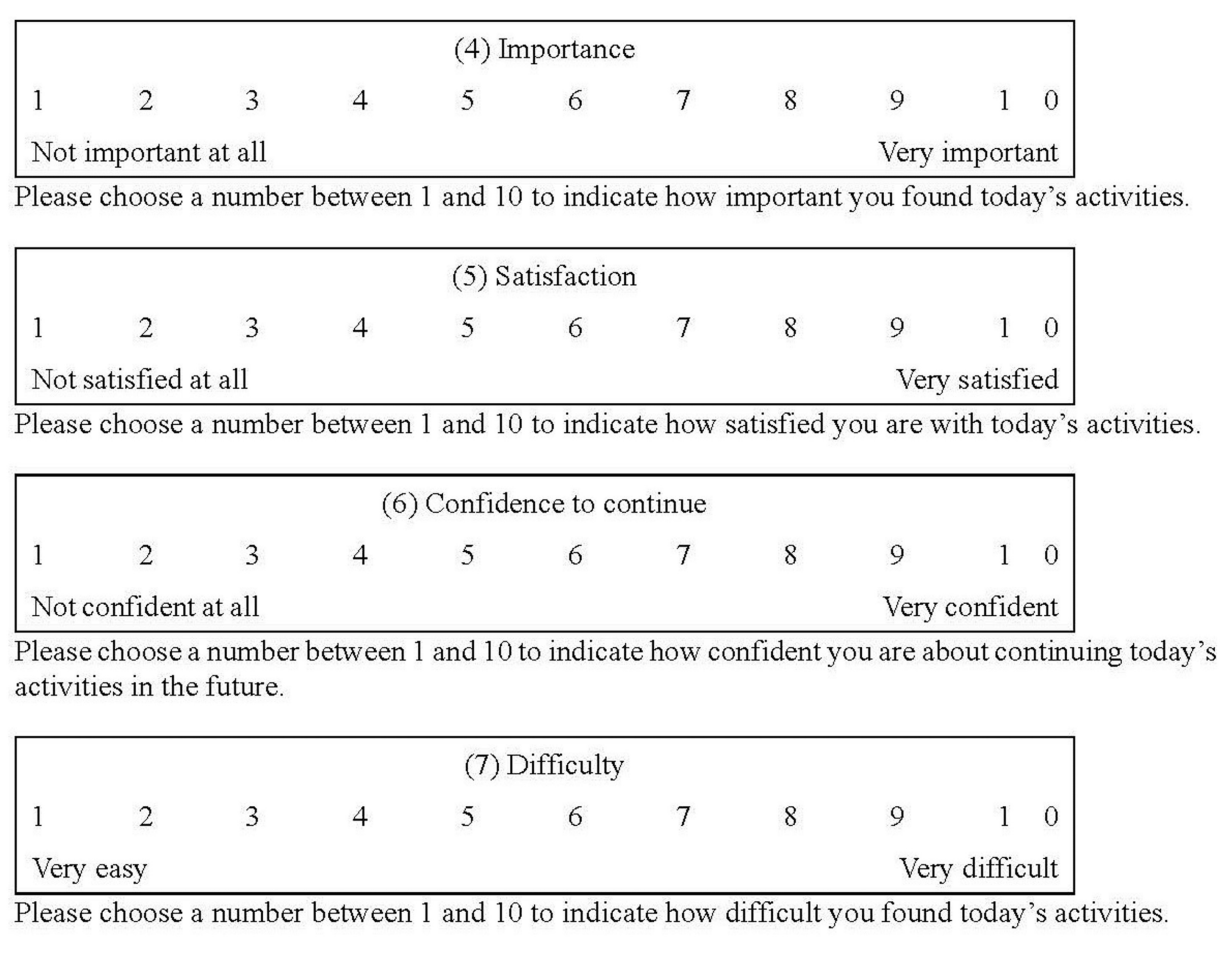
Likert scale included in the goal-setting diary The participants daily recorded their scores in sections (4)-(7) of the goal-setting diary, while referencing this figure (as shown in Figure 1)

The goal-setting diary consists of two parts: Part 1, which is recorded on the first day, and Part 2, which is completed daily (Figure 1). Part 1 includes a section where users can record their (1) weekly goals. Part 2 includes the following sections: (2) today’s activities to achieve weekly goals, (3) strategy for today’s activities, (4) importance, (5) satisfaction with today’s activities, (6) confidence to continue (confidence in their ability to maintain future activities), and (7) task difficulty. Items (1)-(3) are answered in a free-response format, while items (4)-(7) are rated using a 10-point Likert scale (Figure 2). Furthermore, (8) persistence could be evaluated by counting the number of days recorded by the user in their goal-setting diary.

The rationale for including items (3)-(7) in the goal-setting diary is as follows: in the rehabilitation process, collaborative communication enables clients to believe in their ability to achieve their goals, which promotes commitment. Commitment consists of importance, confidence to continue, and satisfaction [4,5,9]. These factors contribute to enhanced performance [5]. Therefore, (4) importance, (5) satisfaction, and (6) confidence to continue were incorporated into the goal-setting diary. Additionally, previous studies [10,11] showed that strategies for achieving goals and the degree of difficulty affected performance. Consequently, (3) strategies and (7) difficulties were also incorporated into the goal-setting diary.

Procedure and Survey Items

We held a research orientation for the OTS, explaining the purpose of this study and the procedure for completing the goal-setting diary. The orientation was scheduled during the class period when no clinical practice was being conducted, because the behavior of OTS toward goal-setting may vary across class periods, clinical practice, and long vacation breaks. OTS who agreed to participate in this study filled out the goal-setting diary daily for seven days, beginning from the orientation date. Upon completion, they submitted their diaries via a designated collection box.

If the OTS had no goal-related activity on a given day, they recorded “nothing” in (2) today’s activities to achieve weekly goals and (3) strategy for today’s activities, while marking 0 points in (4)-(7) in the goal-setting diary. Regarding (3), the total number of strategies recorded by the OTS was used as data. For example, in Figure 1, two strategies were recorded on Day 1. If the OTS used two strategies daily, there would be 14 strategies over seven days. Conversely, if no strategy was employed on a given day, the OTS would record “nothing.” Additionally, (4)-(7) were converted into data based on the total score for the seven days (score range: 0-70). Furthermore, (8) persistence was converted into a numerical score (range 0-7) based on the number of days recorded by the OTS.

**Figure 3:**
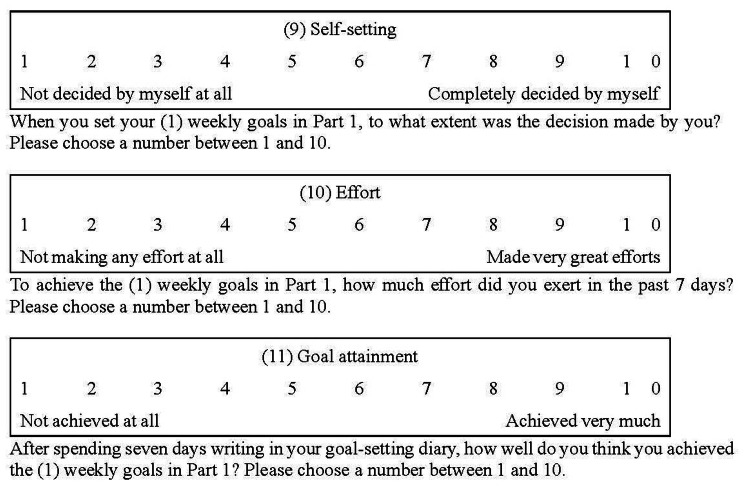
Likert scale used to collect data for explanatory variables used in this study The participants recorded their scores on the day they completed the goal-setting diary

Notably, the goal-setting diary did not include other factors (such as self-setting and effort) that are supposed to influence performance according to the goal-setting theory. Self-setting refers to the extent to which the OTS determined their (1) weekly goals by themselves during the goal-setting. This was not included in the goal-setting diary because there was no need for individuals to monitor daily whether their goals were self-set. Effort was not included because, in an occupational therapy program for a survivor of cancer that utilized a goal-setting diary [6], monitoring effort involved the risk of overwork when the survivor filled in the goal-setting diary every day as homework. Consequently, in this study, (9) self-setting and (10) effort, which were not included in the goal-setting diary, were evaluated using a 10-point Likert scale (Figure 3). Similarly, (11) goal attainment, the objective variable, was assessed. These scores ranged from 1 to 10 points because the OTS recorded them only after the goal-setting diary was completed.

Processing of Missing Values

To address missing values, we employed a reexamination method. Specifically, if the OTS submitted a goal-setting diary with incomplete entries, we promptly requested that they provide the missing data.

Statistical analysis

The (11) goal attainment was used as the objective variable, and items (3)-(10) (number of strategies, importance, satisfaction, confidence to continue, difficulty, persistence, self-setting, and effort) and dummy variables for training institutions and sex were used as explanatory variables. The training institutions were dummy-coded as a university and others (junior college and vocational school).

First, we confirmed the normality of the objective and explanatory variables using the Shapiro-Wilk test. Following this, Spearman’s rank correlation coefficient was calculated to perform a correlation analysis between the explanatory variables, and variables with a value of |r| > 0.8 were excluded.

Second, a multiple regression analysis (stepwise method) was conducted. Variables with a variance inflation factor (VIF) of ≥5 [12] were considered multicollinear. All analyses were performed using IBM SPSS Statistics version 29 (IBM Corp., Armonk, NY). The p value of <0.05 was considered statistically significant.

Ethical considerations

Before its commencement, this study was approved by the Research Ethics Committee of the Prefectural University of Hiroshima (approval number: issue 24MH012). Participants were informed in advance that they could decide for themselves whether to attend the orientation for this study and complete/submit the goal-setting diary. They were provided with both written and verbal explanations that their privacy would be protected, they could withdraw their consent at any time, even after agreeing to participate, and their decision to participate would have no impact on their academic grades at the training institution. All participants subsequently provided their written consent before engaging in the study, and we confirm that no identifying information about the participants is included in the article.

## Results

A total of 167 OTS (24 men; average age 20 years; standard deviation, SD = 1.8; 93 university students, 34 junior college students, and 40 vocational school students) completed and submitted a goal-setting diary, resulting in a response rate of 66.3%. Data were recollected from seven participants (4.2%) with missing values.

The OTS set weekly goals in their goal-setting diaries, such as going to bed early and waking up early, studying for qualifications, cleaning their homes daily, reading purchased books, and cooking at least once daily. Additionally, most of them filled out their goal-setting diaries for at least five of the seven days. Descriptive statistics for the objective and primary explanatory variables are presented in Table 1. Among the participants, 105 OTS (62.9%) reported a self-setting score of 10 points, which was dummy-varied into two values of 10 and 1-9 points.

**Table 1:** Descriptive statistics for the objective variable and explanatory variables (excluding sex and training institution) ^a^Scores range: 0-70 ^b^Scores range: 0-7 ^c^Scores range: 1-10

Variables	Value, median (first-third quartile)
Number of strategies	4 (2-5)
Importance^a^	40 (32-51)
Satisfaction^a^	40 (31-48)
Confidence to continue^a^	31 (22-40.5)
Difficulty^a^	26 (18-34)
Persistence^b^ (number of days recorded)	5 (5-7)
Self-setting^c^	10 (8-10)
Effort^c^	7 (5-8)
Goal attainment^c^	7 (6-8)

Spearman’s rank correlation coefficient was calculated, and no variables exhibited |r| > 0.8. Therefore, all variables were included in the multiple regression analysis. As a result of stepwise multiple regression analysis, the analysis of variance table showed a significant difference (p < 0.001). The (10) effort and (5) satisfaction were identified as significant explanatory variables (Table 2). The VIF for all variables was <5. The adjusted R^2^ value was 0.57, suggesting the model had substantial predictive power. The Durbin-Watson statistic was 1.9, which was close to 2, suggesting independence of residuals. In addition, residual analysis using the Shapiro-Wilk test showed that residuals were normally distributed (p = 0.30). An OTS (0.6%) showed an outlier value that exceeded ±3 SD of the predicted value compared to the actual measured value.

**Table 2:** Stepwise multiple regression analysis for goal attainment (n = 167) Adjusted R2 = 0.57 ^*^p < 0.05 B: partial regression coefficient; SE: standard error; β: standardized partial regression coefficient; CI: confidence interval; VIF: variance inflation factor

Model		B	SE	β	t-value	p value	B (95% CI)	VIF
1	Constant	1.93	0.39	None	5.10	<0.001^*^	1.21-2.74	None
	Effort	0.74	0.06	0.72	13.21	<0.001^*^	0.63-0.85	1.00
2	Constant	1.28	0.39	None	3.23	<0.001^*^	0.50-2.06	None
	Effort	0.58	0.06	0.56	9.16	<0.001^*^	0.46-0.71	1.43
	Satisfaction	0.05	0.01	0.28	4.60	<0.001^*^	0.03-0.06	1.43

## Discussion

This foundational study explored the relationship between the contents of the goal-setting diary and goal attainment. Multiple regression analyses revealed that effort and satisfaction were significantly associated with goal attainment. Furthermore, it is possible to predict goal attainment based on effort and satisfaction because these analyses demonstrated a high degree of model fit. However, the fact that a participant (0.6%) showed an outlier value exceeding ±3 SD, which occurs at a probability of approximately 0.2%, should be interpreted carefully. This participant set a weekly goal of “reading 10 articles,” and his/her recording date was three days, and the goal attainment was 10 points. In these three days, he/she read one, two, and 21 articles, respectively. Namely, he/she concentrated on the goal for only one of the seven days. Therefore, this is an outlier value because the goal attainment score was high, although the total scores of (5) satisfaction and (10) effort for seven days were relatively low. Consequently, our goal-setting diary can be utilized as a tool related to goal attainment if effort is regularly invested within a seven-day period. The results support the face validity of the goal-setting diary, as they are consistent with the original purpose of this tool, which is to integrate daily self-monitoring into a rehabilitation program.

This study was conducted with the larger aim of applying a goal-setting diary to rehabilitation practice. However, our results should also be interpreted carefully, as participants were healthy OTS. The following discussion describes the consistency between this study and previous studies, which reported that effort and satisfaction levels affect goal achievement in clinical settings. In addition, we discuss strategies and highlight key points when using a goal-setting diary in clinical settings.

Effort

The goal-setting theory suggests that greater effort is expended when goals are specific and challenging [4]. Specifically, increased effort is a key factor that contributes to higher performance [4,13]. In post-acute brain injury rehabilitation, it has been reported that clinician-rated client effort is associated with outcomes such as physical/cognitive abilities, emotional/interpersonal adjustment, community participation, and level of supervision required [14]. This study demonstrated the relationship between effort and goal attainment, consistent with previous reports. Therefore, the participants who achieved higher levels of goal attainment during the seven-day goal-setting diary period likely exerted more effort by setting specific and challenging goals on the first day.

When clients are expected to complete a goal-setting diary, practitioners should be careful. For example, stressful experiences impair efforts to be physically active [15]. In addition, clients experiencing depression often face challenges owing to their obsessive personalities and meticulousness [16]. Therefore, continuous recording of the goals and encouragement to exert effort may be counterproductive, potentially increasing stress and reducing motivation. In such cases, clients should not be asked to make excessive efforts. Thus, occupational therapists should educate clients sufficiently and monitor how clients are using their goal-setting diary before allowing the transition to the client self-monitoring phase. Alternatively, the goal-setting diary can be used to monitor the degree of effort so that it does not exceed a certain level. This will help prevent clients' overwork.

Satisfaction

The goal-setting theory suggests that satisfaction is not a factor influencing performance but rather a result of performance [4]. Similarly, occupational therapy programs that incorporate goal-setting, which focus on occupations with clients who have undergone high tibial osteotomy or have mental health disorders, have reported improved satisfaction with the results of approaching their goals [17,18]. However, a study demonstrated that goals could be consistently pursued without deviation by regularly monitoring satisfaction with goal-related behaviors during an occupational therapy program for patients with cancer [19]. Therefore, satisfaction with the final goal as an outcome and satisfaction with daily activities related to the final goal need to be distinguished. This study revealed that daily monitoring of satisfaction was significantly associated with the degree of goal attainment on the final day of completing the goal-setting diary.

A challenge of this study is that collaboration between individuals and therapists was not examined because the participants engaged with goals by themselves. A qualitative study involving patients with neurological disorders reported that commitment to goals and satisfaction increased when there was a high level of collaboration between patients and therapists [9]. In addition, commitment and satisfaction are moderators of performance, suggesting that collaboration has the potential to enhance goal attainment [5]. Therefore, fostering collaboration between patients and therapists could effectively increase satisfaction with daily activities, ultimately contributing to goal achievement. Hence, it is recommended that the goal-setting diary be integrated into a clinical setting based on collaboration between patients and therapists while monitoring satisfaction.

Limitations and future directions

This study has some limitations. The findings are limited by the short duration of data collection, which was restricted to seven days. Therefore, the results cannot conclusively establish causal relationships over medium- to long-term timelines. In addition, since the data were collected outside the context of clinical rehabilitation, outcomes may differ if the data were gathered from clients in clinical settings. Consequently, future studies should involve clients in clinical settings.

This study primarily aimed to develop a goal-setting diary that includes variables relevant to goal attainment and has a simple format for future clinical application. Of the factors that showed a significant relationship with goal attainment, effort was not recorded by the participants every day. Therefore, it is possible to revise the goal-setting diary to a format in which effort and satisfaction are recorded daily. This revision would not only improve the potential for goal attainment through continuous monitoring of effort and satisfaction levels but also enhance the diary’s usability and ease of completion.

## Conclusions

This study demonstrated that effort and satisfaction are significantly related to goal attainment. The results from our study contribute to the development of a simple and effective goal-setting diary that integrates self-monitoring of two important factors: effort and satisfaction, toward achieving goals. However, this result may only be obtained under the condition that effort is exerted regularly during the recording period. Further research is needed to test the revised goal-setting diary in clinical settings, allowing for a broader application and validation of its effectiveness.
